# Structural Assessment of *Chlamydia trachomatis* Major Outer Membrane Protein (MOMP)-Derived Vaccine Antigens and Immunological Profiling in Mice with Different Genetic Backgrounds

**DOI:** 10.3390/vaccines12070789

**Published:** 2024-07-18

**Authors:** Shea K. Roe, Tianmou Zhu, Anatoli Slepenkin, Aym Berges, Jeff Fairman, Luis M. de la Maza, Paola Massari

**Affiliations:** 1 Department of Immunology, Tufts University School of Medicine, Boston, MA 02111, USA; shea.roe@tufts.edu (S.K.R.);; 2 Department of Pathology and Laboratory Medicine, University of California, Irvine, CA 92697, USAlmdelama@uci.edu (L.M.d.l.M.); 3 Vaxcyte Inc., 825 Industrial Road, Suite 300, San Carlos, CA 94070, USAjeff.fairman@vaxcyte.com (J.F.)

**Keywords:** vaccine, *Chlamydia trachomatis*, MOMP, antibodies, B-cell epitopes

## Abstract

*Chlamydia trachomatis* (*Ct*) is the most common cause of bacterial sexually transmitted infections (STIs) worldwide. *Ct* infections are often asymptomatic in women, leading to severe reproductive tract sequelae. Development of a vaccine against *Chlamydia* is crucial. The *Chlamydia* major outer membrane protein (MOMP) is a prime vaccine antigen candidate, and it can elicit both neutralizing antibodies and protective CD4+ T cell responses. We have previously designed chimeric antigens composed of immunogenic variable regions (VDs) and conserved regions (CDs) of MOMP from *Chlamydia muridarum* (*Cm*) expressed into a carrier protein (PorB), and we have shown that these were protective in a mouse model of *Cm* respiratory infection. Here, we generated corresponding constructs based on MOMP from *Ct* serovar F. Preliminary structure analysis of the three antigens, PorB/VD1-3, PorB/VD1-4 and PorB/VD1-2-4, showed that they retained structure features consistent with those of PorB. The antigens induced robust humoral and cellular responses in mice with different genetic backgrounds. The antibodies were cross-reactive against *Ct,* but only anti-PorB/VD1-4 and anti-PorB/VD1-2-4 IgG antibodies were neutralizing, likely due to the antigen specificity. The cellular responses included proliferation in vitro and production of IFN-γ by splenocytes following *Ct* re-stimulation. Our results support further investigation of the PorB/VD antigens as potential protective candidates for a *Chlamydia* subunit vaccine.

## 1. Introduction

*Chlamydia trachomatis* (*Ct*) is the most common bacterial sexually transmitted infection worldwide, with an estimated 131 million new cases yearly [1,2]. There are 15 major *Ct* serovars, classified based on variants of the major outer membrane protein, MOMP [3]. Serovars D through K are responsible for genital, respiratory, gastrointestinal and ocular infections, serovars A, B, Ba and C for trachoma, and serovars L1–L3 for lymphogranuloma venereum [4]. Men infected with *Ct* typically develop acute clinical manifestations, including urethritis and epididymitis, while symptoms are often absent in infected women (although cervicitis can be reported) [5]. The asymptomatic nature of this disease in women can not only lead to increased transmission, but also, most importantly, delays in treatment; untreated infections and disease progression cause long-term sequelae, such as pelvic inflammatory disease (PID), ectopic pregnancy and infertility. Moreover, neonatal conjunctivitis and pneumonia are reported due to mother-to-newborn transmission, and *Ct* is also a risk factor for contracting other STIs [6]. Once infection has been diagnosed, antibiotic therapy is available, but it does not prevent recurring infections, and concerns have been raised about its effect on the development of natural immunity [7,8,9]. A vaccine against *Chlamydia* remains the best approach to control this pathogen and reduce its recurrence, transmission and disease prevalence, but the development of a protective vaccine remains a global challenge [10,11].

Initial efforts with a whole organism-based vaccine (live or inactivated) only showed limited protection against trachoma; a short-lived and serovar/subgroup-specific immune response was achieved in humans and non-human primates but, in some individuals, this was accompanied by hypersensitivity reactions and increased inflammation after re-exposure to *Ct* [12,13]. Second-generation subunit vaccines based on chlamydial proteins, i.e., polymorphic membrane proteins (Pmps) [14], the chlamydial-protease-like activity factor (CPAF) protein [15], and MOMP [16], have been explored preclinically. MOMP has shown induction of the best protective immunity responses in several animal models [17,18,19]. Third-generation DNA-based vaccines also have been examined, with relatively little success [20,21].

MOMP, a trimeric porin, is the most abundant surface-exposed protein in *Chlamydia* (~60% of the outer membrane protein mass); the structure of MOMP has not been solved but its topology model indicates a putative 16-stranded β-barrel transmembrane core region with eight surface-exposed loops and eight short periplasmic turns per monomer [22,23,24]. Loops 2, 3, 5 and 7 contain regions of sequence variability (variable domains, VDs) flanked by conserved sequence regions (constant domains, CDs). Both the VD and the CD regions encompass B-cell and T-cell epitopes (known and predicted) that predominantly induce neutralizing antibodies and protective T-cell immunity, respectively [25,26,27]. Antibodies are protective against reinfection, and MHC class II-restricted IFN-γ-producing CD4+ T-cells are protective against primary and secondary infections [28,29,30]. Preclinical vaccine studies with purified MOMP and a variety of adjuvants have shown robust immune responses and protection against *Chlamydia* genital and respiratory challenges in mice [31,32,33,34,35,36,37] and against ocular infection in monkeys [18]. Unfortunately, a MOMP-based vaccine presents challenges for scaling up production of this cysteine-rich protein in its native form, and obtaining a recombinant form of MOMP that is correctly folded and retains conformational epitopes has not been successful so far. The current best alternative to an immunologically active recombinant MOMP is CTH522, a recombinant antigen composed of a large sequence of MOMP encompassing CD regions followed by several repeats of the VD4 of four different genital *Ct* serovars (D, E, F, and G), thus encompassing both T-cell and B-cell epitopes [28,38]. This synthetic antigen has shown promising results with CAF01 as an adjuvant and has completed Phase I clinical trials [39,40,41].

In previous work, we have designed MOMP-based chimeric antigens composed of entire loops of MOMP expressed into the structurally similar protein PorB from *Neisseria lactamica* as a carrier [42]. In a proof-of-concept study using the *Chlamydia muridarum* (*Cm*) MOMP, we have shown that three hybrid constructs containing different combinations of *Cm* MOMP loops were protective in a mouse model of *Cm* respiratory infection [43]. We have generated equivalent constructs based on the corresponding regions of MOMP from *Ct* serovar F. Here, we present an initial evaluation of the structural features of these antigens and their ability to induce *Ct*-specific immune responses in mice with different genetic backgrounds. Our results support the potential of these hybrid antigens for inducing robust humoral and cellular responses against *Ct* and for a future inclusion in a *Chlamydia* vaccine.

## 2. Materials and Methods

**Bioinformatics analyses.** The PorB/VD constructs were designed based on analysis of the DNA and protein sequences of the *N. lactamica* strain Y92-1009 PorB (UniProt accession number: B2BFC2) [42] and of MOMP (*Ct* serovar F) (UniProt accession number: P16155) [44] as references. Identification of suitable residues for loop swapping between PorB and MOMP was carried out as previously described [42].

**Cloning, expression, and protein purification.** Cloning of PorB/VD chimeric proteins was outsourced to Genscript (Genscript, Piscataway, NJ, USA) and was performed by gene synthesis and sub-cloning. The PorB/VD1-3, PorB/VD1-4 and PorB/VD1-2-4 constructs were designed based on our previous strategy for generating *Cm* MOMP-based constructs [42,43]. The following constructs were created: PorB/VD1-3, in which PorB loops 5 and 7 were replaced with *Ct* serovar F MOMP loop 2 (containing VD1) and loop 5 (VD3), respectively; PorB/VD1-4, in which MOMP loop 2 (VD1) and loop 7 (VD4) replaced PorB loops 5 and 4, respectively; and PorB/VD1-2-4, in which PorB loops 5, 6 and 4 were replaced by MOMP loop 2 (VD1), loop 3 (VD2) and loop 7 (VD4), respectively. pET30a plasmids encoding for the recombinant PorB/VDs were transformed into *E. coli* BL21 (DE3) and plated on LB agar plates containing kanamycin (50 µg/mL) as a selection antibiotic. Single colonies were picked and inoculated in LB liquid culture with kanamycin (50 µg/mL). The presence of the *porB/VD* genes was confirmed by DNA extraction (Miniprep kits, Qiagen, Hilden, Germany), digestion with NdeI and HindIII and 2% agarose (m/vol) gel electrophoresis. For the protein expression and purification, liquid cultures were grown overnight in LB with kanamycin as above, diluted to O.D._600_ = 0.6–0.8 and induced with IPTG (0.5 mM final concentration) for 4 h at 37 °C, while inclusion bodies were isolated and protein purification was carried out by column chromatography as previously described [42]. Purification of recombinant PorB and of *Ct* serovar F MOMP were carried out as previously described [36,42].

**Structure modeling and predictions.** The predicted model of PorB was generated with SWISS MODEL Works [45] and AlphaFold v2.0 [46] based on the 3.2 Å resolution X-ray crystal structure of the *Neisseria meningitidis* PorB (PDB ID 3WI4) [47]. The PorB model was used to subsequently build the PorB/VD1-3, PorB/VD1-4 and PorB/VD1-2-4 models. The cartoon representations were rendered with PyMOL [48]. The protein charges were evaluated with the Prot pi|Protein Tool (https://www.protpi.ch (accessed on 11 July 2024)) (version 2.2.29.152). Secondary structure predictions were obtained using the SOPMA server (https://prabi.ibcp.fr/htm/site/web/app.php/home (accessed on 4 April 2024)) [49] with the default settings (output width: 70; similarity threshold: 8; number of states: 3-helix, sheet, coil). For each modeling, at least 25 or more proteins were aligned out of >500 proteins in the sub-database. Linear (continuous) and conformational (discontinuous) B-cell epitope predictions were obtained with ElliPro [50] with standard cut-offs.

**Gel electrophoresis.** Purified PorB and PorB/VDs were examined by electrophoresis on 12% polyacrylamide gels in the presence of SDS or in the absence of SDS (modified PAGE) in both the acrylamide gel and loading buffer (Bio-Rad, Hercules, CA, USA), as previously described [42]. For complete denaturation, proteins in SDS-containing loading buffer were also incubated at 100 °C for 5 min prior to electrophoresis on SDS-containing gels. Proteins in SDS-free loading buffer were incubated at 25 °C prior to electrophoresis on non-SDS gels. The protein bands were visualized by Coomassie staining.

**SEC-MALS chromatography**. Size exclusion chromatography with multi-angle light scattering (SEC-MALS) chromatography was performed using an Agilent 1200 HPLC system with a UV detector, a HELEOS 18-angle light-scattering detector and an Optilab T-rEX refractive index detector (Wyatt Technology Corporation, Santa Barbara, CA, USA) at 25 °C on a Superdex 200 column 10/300 equilibrated and run with PBS/0.05% Zwittergent 3–14 (pH 7.5) at a flow rate of 0.4 mL/min [51]).

**Immunization of mice.** Female BALB/c mice and C57Bl/6 mice (4–6 weeks old, Jackson Laboratory, Bar Harbor, ME, USA) were housed and cared for per protocols approved by the National Institutes of Health (NIH) and Tufts University IACUC. The mice (n = 5 per group) were immunized three times at three-week intervals with the purified proteins (10 µg each) using Alum (Imject, 40 mg/mL aluminum hydroxide, 40 mg/mL magnesium hydroxide) (Thermo Fisher Scientific, Waltham, MA, USA) (1:1 *v*/*v* ratio, as specified by the manufacturer) and monophosphoryl lipid A (MPLA) (10 µg/mouse/dose) (Avanti Lipids, Alabaster, AL, USA) as adjuvants, in a final volume of 150 µL. The immunizations were delivered subcutaneously (50 µL) and intramuscularly (50 µL in each quadricep). An additional group of mice was immunized with the adjuvant alone. Preimmune sera were collected prior to the first immunization, and immune sera two weeks after immunization (wk. 2, wk. 5 and wk. 8) by sub-mandibular bleed. At the end of the immunization schedule, vaginal lavages were also collected (wk. 8). Sera and lavages were stored at −80 °C until use.

**Antibody ELISA.** The ELISA plates were coated with 100 µL of purified proteins (2 μg/mL) or with *Ct* serovar F elementary bodies (EBs) (1 µg/well) as previously described [16,42]. The plates were blocked with 2% bovine serum albumin (BSA) in PBS/0.02% Tween-20 and incubated with serial dilutions of pooled mouse sera or vaginal lavages, followed by incubation with AP-conjugated secondary anti-mouse total IgG, IgG1, IgG2a, or IgG2c antibodies (Southern Biotech, Birmingham, AL, USA) and one-step PNPP substrate (Pierce) at O.D._405_ were used for detection as specified by the manufacturer. The total IgG and IgG subclasses were quantified in the pooled sera (µg/mL ± SD) or pooled vaginal lavages (ng/mL ± SD) from triplicate or quadruplicate wells using antibody reference standard curves (Southern Biotech) and a linear regression function. The IgG subclass ratios were calculated as IgG2a/IgG1 (BALB/c) or IgG2c/IgG1 (C57Bl/6).

**Cytokine ELISA.** The serum levels of Th1-type cytokines IFN-γ and IL-12p70, Th2-type cytokines IL-4 and IL10, and inflammatory cytokines IL-6, TNF-α (Opt-EIA kit, BD Biosciences, Franklin Lakes, NJ, USA) and IL-1β (BioLegend, San Diego, CA, USA) were measured by ELISA according to the manufacturers’ protocol. The pooled sera were tested in triplicate, while the cytokine levels were quantified in pg/mL, normalized to the levels in sera from mice immunized with the adjuvant only and expressed as ratio ± SD. IFN-γ and IL-4 were also measured in supernatants from the splenocytes as described below; the cytokines were quantified in pg/mL from two independent experiments in triplicate wells and expressed as the ratio to the negative control (medium alone) ± SD.

**Stocks of *C. trachomatis*.** The *Ct* serovar F (strain IC-Cal-3; ATCC VR-346, Manassas, VA, USA) was grown in HeLa-229 cells as previously described [52]. *Ct* EBs were purified and stored at −80 °C in sucrose phosphate-glutamate buffer.

***Chlamydia* in vitro neutralization assay.** For the in vitro neutralization assay, mouse sera were serially diluted two-fold in Ca^+2^/Mg^+2^-free PBS containing 5% guinea pig serum as a source of complement in duplicate as previously described [53]. The sera were then incubated with *Ct* serovar F (10^4^ inclusion forming units (IFUs)) for 45 min at 37 °C, followed by centrifugation onto HeLa-229 monolayers previously grown in flat-bottom 96-well plates. The cell monolayers were incubated in medium containing cycloheximide (1 µg/mL) for 40 h, then fixed with methanol and incubated with the mAb E4 that recognized the VD4 of MOMP for staining and counting of the chlamydial IFUs. Neutralization was defined as a ≥50% decrease in the number of IFUs compared with the controls incubated with sera from naïve mice.

**Splenocytes proliferation assay**. Spleens were collected from the immunized mice, pooled, and whole splenocyte suspensions were obtained as previously described [54]. The splenocytes (5 × 10^5^ cell/mL) were seeded in 96-well flat-bottom plates in 100 μL of RPMI medium containing 10% fetal bovine serum (FBS), 100 U/mL penicillin and 100 μg/mL streptavidin, 2 mM L-glutamine and 20 mM HEPES (Sigma, St. Louis, MO, USA), pH 7.0, in triplicate wells. The cells were stimulated with *Ct* EBs (10 μg/mL), concanavalin A (ConA, positive control) (10 μg/mL) or with medium alone (negative control) for 72 h in a 5% CO_2_ incubator. Proliferation was determined using the MTT (3-[4,5-dimethylthiazol-2-yl]-2,5 diphenyl tetrazolium bromide) incorporation assay (Millipore Sigma, Burlington, MA, USA) per the manufacturer’s specifications. Absorbance was measured spectrophotometrically at O.D._570_ with an O.D._690_ reference wavelength. The cell proliferation was expressed as the Stimulation Index [(cell proliferation in the stimulated wells/cell proliferation in the control wells) × 100]. Supernatants were collected from additional sets of triplicate wells for measurement of the secreted IFN-γ and IL-4 by ELISA, as described above.

**Statistical analyses.** Statistical significance was evaluated with GraphPad Prism v10.1.2 by one-way or two-way analysis of variance (ANOVA) using Tukey’s multiple comparisons test or Sidak’s multiple comparisons test. Differences below *p* value = 0.05 were considered significant and are indicated as follows: * *p* = 0.05; ** *p* = 0.005; *** *p* = 0.0005 and **** *p* < 0.0001.

## 3. Results

### 3.1. Sequence Analysis of C. trachomatis Serovar F MOMP and N. lactamica Y92-1009 PorB

**Figure 1:**
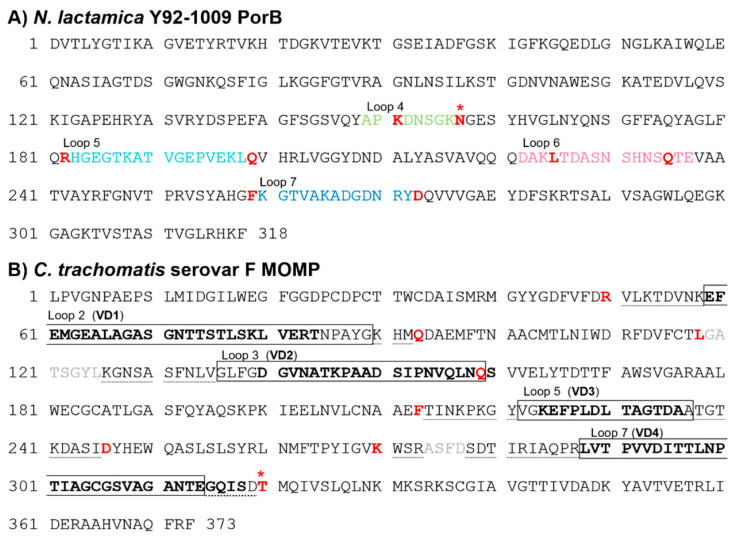
Amino acid sequences of PorB and MOMP. (**A**) *N. lactamica* Y92-1009 PorB (UniProt accession number B2BFC2). Loop 4: green. Loop 5: teal. Loop 6: pink. Loop 7: blue. Anchor residues: bold red. N176: red star. (**B**) *Ct* serovar F MOMP (UniProt accession number: P16155). Loops: boxed residues. VDs, bold residues. CDs and turn residues included in loop swapping: dotted underlined and gray, respectively. Anchor residues: bold red. T342: red star.

We have previously utilized PorB as a carrier for the loops of *Cm* MOMP and identified PorB loops 4, 5, 6, and 7 as suitable for replacement with the MOMP loops [42]. We utilized the same strategy to generate hybrid *Ct* serovar F MOMP-based constructs. The amino acid sequence of PorB is shown in Figure 1A, with loops 4, 5, 6 and 7 indicated in color along with the residues used as anchors for loop swapping. Figure 1B shows the amino acid sequence of the *Ct* serovar F MOMP. The MOMP regions transferred into PorB included loops 2, 3, 5 and 7 (Figure 1B, boxed), where the VD residues are bolded, and loop-flanking residues extending into the CDs (Figure 1B, dotted underlined). In the absence of an exact amino acid match for asparagine 176 (N176) in PorB loop 4, threonine 342 (T342) in MOMP loop 7 was chosen as the anchor residue (Figure 1A and Figure 1B, red star, respectively) (both polar and uncharged amino acids).

The following constructs were generated: PorB/VD1-3, PorB/VD1-4 and PorB/VD1-2-4 (Figure S1). For all three constructs, the MOMP sequence encompassing loop 2 containing VD1 replaced the PorB loop 5 (Figure S1, teal). For PorB/VD1-3, the MOMP sequence of loop 5 containing VD3 replaced the PorB loop 7 (Figure S1A, blue); for PorB/VD1-4, the MOMP sequence of loop 7 containing VD4 replaced the PorB loop 4 (Figure S1B, green), and for PorB/VD1-2-4, PorB loops 5 and 4 were replaced as above, and the MOMP sequence of loop 3 containing VD2 was used to replace the PorB loop 6 (Figure S1C, pink).

### 3.2. PorB/VDs Sequence Analysis and Structure Predictions

**Figure 2:**
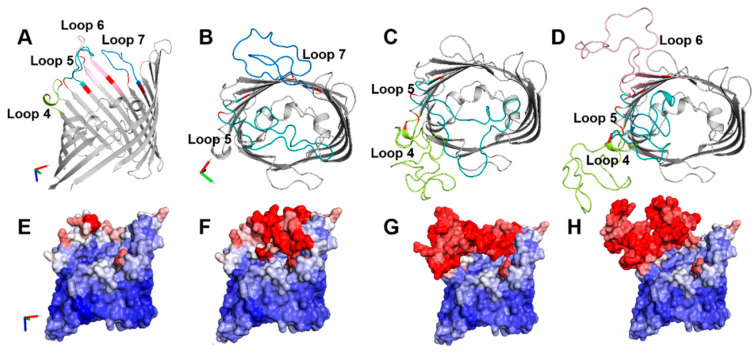
Structure predictions. (**A**) Cartoon model of the predicted monomer of PorB (side view) based on the crystal structure of *N. meningitidis* PorB (PDB ID 3WI4). Loop 4, green. Loop 5, teal. Loop 6, pink. Loop 7, blue. Anchor residues, red. Cartoon models of the predicted monomer of (**B**) PorB/VD1-3 (top view). Loop 5, teal. Loop 7, blue, (**C**) PorB/VD1-4. Loop 4, green. Loop 5, teal, and (**D**) PorB/VD1-2-4. Loop 4, green. Loop 5, teal. Loop 6, pink. (**E**–**H**) Cartoon models above colored by predicted B-factor (side view). Red: predicted areas of thermal motion and loop flexibility. Blue: immobile. The x-y-z axis indicates the model’s orientation.

To examine whether the PorB/VD hybrid proteins retained the core structural properties of the carrier protein PorB, several bioinformatics tools were used. Figure 2A shows a cartoon model of PorB based on the crystal structure of the *N. meningitidis* PorB (PDB ID 3WI4) previously solved by our group [47]. Analysis of the protein charges, as calculated from the amino acid sequence, indicated a total charge of +9 (+42 positively charged residues/−33 negatively charged residues) and a net charge at pH 7.4, (z), of +0.916. The local loop charges were +1 (+2/−1) for loop 4, +1 (+4/−3) for loop 5, −1 (+2/−3) for loop 6, and +1 (+3/−2) for loop 7.

Figure 2B shows a cartoon model of the predicted PorB/VD1-3 monomer, with the swapped loops in color to facilitate visualization. The total charge of this protein was also +9 (+46/−37), with (z) = +0.899. The impact of the loop replacement on the local charges is shown in Table 1.

For PorB/VD1-3, replacement of PorB loops 5 and 7 with MOMP loop 2 (VD1) and MOMP loop 5 (VD3), respectively, led to local charge changes from +1 to +2 (loop 5) and from +1 to −1 (loop 7), respectively (Table 1). This could lead to electrostatic surface charge interactions among these loops and may have a potential impact on their orientation and/or surface exposure compared to the original PorB. PorB/VD1-4 (Figure 2C) had a total charge of +8 (+47/−39), with (z) = −0.204; the same charge changes from +1 to +2 occurred for PorB loop 5 (swapped with MOMP loop 2 (VD1) as above) and from +1 to −1 for PorB loop 4, swapped with MOMP loop 7 (VD4) (Table 1). This charge differential may have similar implications for the electrostatic interactions among these loops as for PorB/VD1-3. The total charge of PorB/VD1-2-4 (Figure 2D) was +8 (+48/−40), with (z) = +0.739. Here, in addition to the charge changes due to the replacement of PorB loops 4 and 5 as above, the local charge of PorB loop 6 changed from +1 to 0 due to replacement with MOMP loop 3 (VD2) (Table 1), implying further electrostatic interactions among the three swapped loops, and maybe also nearby loops. A coloring of the predicted structure model cartoons by the B-factor (a parameter that describes the predicted spatial positioning and dynamics compatibility of a protein as thermal motion and loop flexibility [55,56]) suggested that the replaced loop regions likely had high mobility and position uncertainty (Figure 2E–H). Secondary structure predictions were carried out with SOPMA (self-optimized prediction method with alignment) [49], a tool that evaluates the presence and percent of α-helix, β-sheet and coil forms. Based on this analysis, minimal to no differences in the content of these three-state descriptions were predicted among PorB and the three PorB/VD constructs (Table 2).

**Figure 3:**
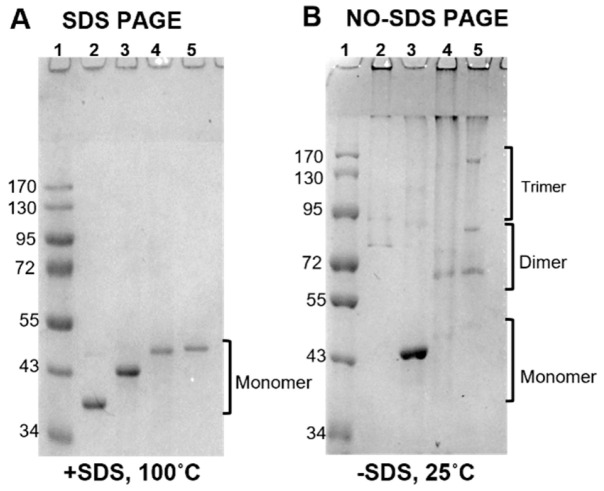
Electrophoretic analysis. (**A**) SDS-PAGE and Coomassie staining. Samples (~2 µg/lane) were resuspended in loading buffer containing SDS and incubated at 100 °C for 5 min for complete denaturation prior to electrophoresis on SDS-containing gel. (**B**) Modified SDS-PAGE and Coomassie staining. Samples were resuspended in SDS-free loading buffer and incubated at 25 °C prior to electrophoresis on SDS-free gel. Lane 1: molecular weight standard. Lane 2: PorB, ~35.7 kDa. Lane 3: PorB/VD1-3, ~38.7 kDa. Lane 4: PorB/VD1-4, ~41.4 kDa. Lane 5: PorB/VD1-2-4, ~44.5 kDa. Bands of molecular weights corresponding to monomers, dimers and trimers are indicated. Higher-order oligomers and aggregates are visible in the stacking gel and at the bottom of the wells in (**B**).

Lastly, the proteins’ electrophoretic behavior was examined in fully denaturing conditions (thermal denaturation and presence of SDS) and in non-denaturing conditions (no thermal denaturation and no SDS). We previously used this method to evaluate the structure of the *Cm* MOMP-based PorB/VD constructs [42]. As shown in Figure 3A, when the proteins were examined by SDS-PAGE in denaturing conditions, only the monomer forms were detected by Coomassie staining of the gel. In non-denaturing conditions, bands corresponding to dimers, trimers and higher-molecular-weight complexes (including large aggregates trapped in the stacking gel wells) were observed for PorB, PorB/VD1-4 and PorB/VD1-2-4 (Figure 3B, lanes 2, 4 and 5), accompanied by the disappearance of the monomer forms. In contrast, a substantial monomer form amount was detected for PorB/VD1-3, even in non-denaturing conditions (Figure 3B, lane 3), with traces of dimers and trimers. Preliminary SEC-MALS analysis [51] supported a monomer ratio of approx. 3 for each construct based on the predicted and observed molecular mass of the proteins [42,57], as deduced by the coupling light scatter (LS) and UV_280_ with elution time (data not shown).

### 3.3. Immunogenicity of PorB/VD Antigens in Mice with Different Genetic Backgrounds

#### 3.3.1. Antibody Responses to Purified Antigens

**Figure 4:**
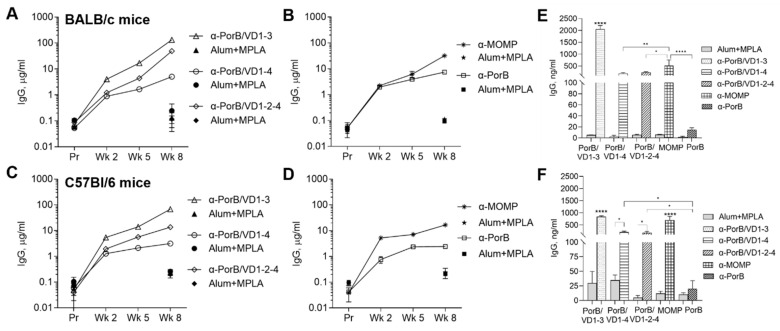
Total IgG antibodies against purified antigens. IgG (μg/mL ± SD) in pooled sera from BALB/c mice immunized with (**A**) PorB/VD1-3 (open triangles), PorB/VD1-4 (open circles), PorB/VD1-2-4 (open diamonds) or (**B**) MOMP (asterisk) and PorB (open squares). Alum+MPLA sera, closed symbols. (**C**,**D**) IgG (μg/mL ± SD) in sera from C57Bl/6 mice as above. Sera were tested in triplicate. (**E**) IgG (ng/mL ± SD) in pooled vaginal lavages from BALB/c mice or (**F**) C57/Bl6 mice immunized with PorB/VD1-3 (dotted bars), PorB/VD1-4 (striped bars), PorB/VD1-2-4 (dashed bars), MOMP (squared bars), PorB (checkered bars) or Alum+MPLA alone (gray bars). Lavages were tested in triplicate. ****, **, and * *p* significant by one-way ANOVA with Tukey’s multiple comparisons test.

To evaluate the contribution of genetic differences to the immune responses to our antigens, the antibody responses were examined in BALB/c and C57Bl/6 mice. BALB/c mice have a predominantly Th2-skewed response and often stronger humoral responses than C57Bl/6 mice (a Th1-dominant response strain) [58,59]. Female mice were immunized with PorB/VD1-3, PorB/VD1-4 or PorB/VD1-2-4 using Alum+MPLA as an adjuvant, and with recombinant *Ct* MOMP, PorB, and adjuvant alone as immunization controls. Sera were collected two weeks after each immunization and the total serum IgG antibodies against each antigen were measured by ELISA. The IgG antibody levels in the preimmune sera from both mouse strains, and in the sera from the adjuvant-only immunized mice, were low (Figure 4A–D, solid symbols). PorB/VD1-3 induced the highest IgG amounts (Figure 4A,C, open triangles) and PorB/VD1-4 the lowest (Figure 4A,C, open circles). Among the controls, higher antibody levels were induced by MOMP than by PorB (Figure 4B,D, asterisks and open squares). A similar trend was observed for the secretory IgGs, with significantly higher anti-PorB/VD1-3 antibody levels in vaginal lavages than anti-PorB/VD1-4 and anti-PorB/VD1-2-4 antibodies (Figure 4E,F). Similarly to the sera, the levels of anti-MOMP antibodies in the vaginal lavages were higher than the anti-PorB antibodies (Figure 4E,F, squared and checkered bars, respectively), and low levels of antigen-specific IgGs were detected in lavages from mice immunized with adjuvant alone (Figure 4E,F, gray bars). These results indicated that the PorB/VD proteins were immunogenic and elicited similar IgG antibody responses in mice with different genetic backgrounds. Based on the IgG levels, PorB/VD1-3 induced a superior IgG response compared to PorB/VD1-4 and PorB/VD1-2-4. Next, the serum IgG subclasses were examined. The Th1:Th2 ratio was calculated as IgG2a (μg/mL)/IgG1 (μg/mL) for BALB/c mice or IgG2c (μg/mL)/IgG1 (μg/mL) for C57Bl/6 mice (Table 3). A stronger Th1-skewed ratio was determined for PorB/VD1-3 and PorB/VD1-4 in C57Bl/6 than in BALB/c mice, in agreement with the Th-bias of these two mouse strains. The Th1:Th2 ratio for PorB/VD1-2-4 was below 1 in both mouse strains, suggesting a strong Th2-skewed antibody response, but there was a statistically significant difference between the C57Bl/6 and BALB/c mice (Table 3). Based on the Th1:Th2 ratio for MOMP and PorB, a slightly more Th-balanced response (ratio closer to 1) was observed. These results suggested that, in addition to the intrinsic Th-bias of the mouse strain, and despite the same adjuvants were being used for all the immunizations (Alum is a Th2-type adjuvant [60] and MPLA favors a more Th1/Th2-balanced response [61]), it is possible that the antigen itself may influence the overall Th antibody skew.

#### 3.3.2. Antigen and Antibody Cross-Reactivity

**Figure 5:**
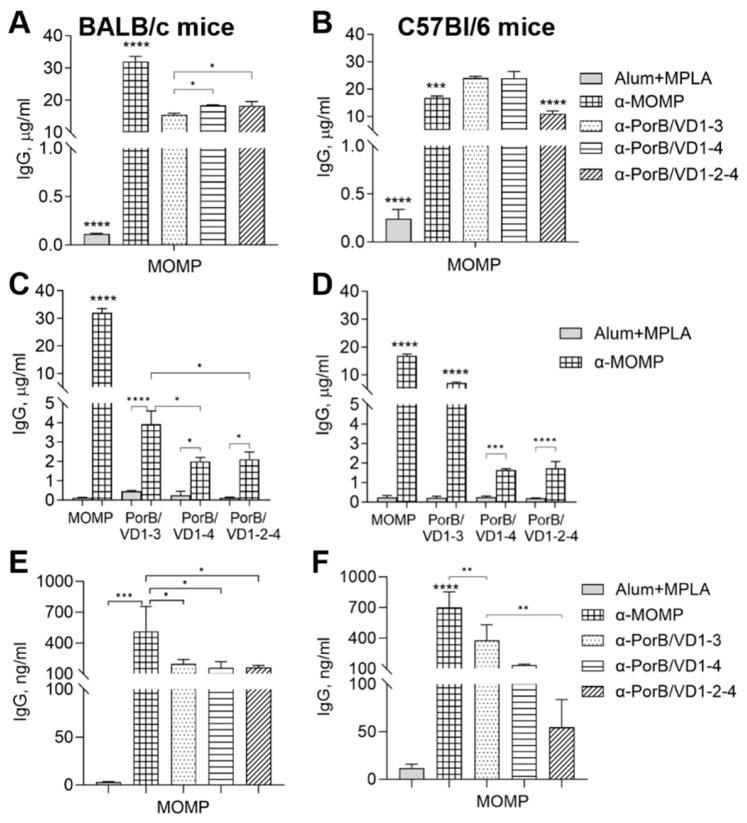
Total IgG antibody cross-reactivity between MOMP and PorB/VDs. IgG (μg/mL ± SD) against MOMP in pooled sera from (**A**) BALB/c mice and (**B**) C57Bl/6 mice immunized with MOMP (squared bars), PorB/VD1-3 (dotted bars), PorB/VD1-4 (dashed bars), PorB/VD1-2-4 (striped bars) or Alum+MPLA (gray bars). Sera were tested in triplicate. (**C**,**D**) IgG against MOMP, PorB/VD1-3, PorB/VD1-4 or PorB/VD1-2-4 in pooled sera from BALB/c mice or C57Bl/6 mice immunized with MOMP (squared bars) or Alum+MPLA (gray bars). (**E**) **I**gG (ng/mL ± SD) against MOMP in pooled vaginal lavages from BALB/c mice and (**F**) C57Bl/6 mice as in (**A**,**B**). Lavages were tested in triplicate. *, **, ***, and **** *p* significant by one-way ANOVA with Tukey’s multiple comparisons test.

The ability of the anti-PorB/VD antibodies to recognize MOMP was examined next. Anti-PorB/VD1-3, anti-PorB/VD1-4 and anti-PorB/VD1-2-4 sera from both BALB/c and C57Bl/6 mice cross-reacted with MOMP (Figure 5A,B), suggesting specificity for the MOMP regions transferred into PorB. Next, the reciprocal cross-reactivity was examined using anti-MOMP mouse sera against the PorB/VD antigens. Figure 5C,D show that PorB/VD1-3, PorB/VD1-4 and PorB/VD1-2-4 were recognized by antibodies in the sera from mice immunized with MOMP, although at a lower extent than MOMP itself. Notably, the anti-MOMP antibodies were most cross-reactive with PorB/VD1-3 (Figure 5C,D). The cross-reactivity of anti-PorB/VD sera against MOMP in vaginal lavages from the immunized mice mirrored that of sera (Figure 5E,F).

To explore whether the observed variability in the antibody responses’ magnitude and antigen cross-reactivity could be due to B-cell epitope differences between the MOMP and the PorB/VD antigens, the epitope prediction tool ElliPro was used [50]. The linear epitope predictions were in agreement with previously described B-cell epitopes [44,62,63]. Table S1 shows the predicted linear B-cell epitope in each MOMP region transferred into PorB. A conformational epitope analysis predicted three discontinuous epitopes in MOMP: a large, surface-exposed epitope composed of residues from all four VDs (Figure S2A), one containing amino acids in the CD regions between loops 3 and 5, between loops 5 and 7, and after loop 7 (not shown), and one encompassing residues in the periplasmic turn region (not shown). The cartoon models in Figure S2B,D show the relative position of the MOMP residues from the conformational epitope region that were transferred into each PorB/VD antigen.

Despite the predictive nature of these models, it would be reasonable to assume that epitope alterations can occur due to the different spatial orientation of the regions involved within the PorB carrier. For example, the residues at the interface of VD1 and VD3 in MOMP (Figure S2A, teal and blue, respectively) were not similarly spatially aligned in PorB/VD1-3 (Figure S2B, dotted teal and blue, respectively). However, with both these regions being predicted to be relatively mobile and oppositely charged (see Table 1), it is possible that they became closer, restoring a VD1–VD3 conformational epitope area more similar to that of the original MOMP. A different situation may occur for PorB/VD1-4: while the VD1 residues in the MOMP VD1-VD4 epitope region (Figure S2A, green and teal, respectively) were in an area of high predicted mobility in PorB/VD1-4 (Figure S2C, dotted teal), the corresponding VD4 residues were directly adjacent to the β-barrel on one end of PorB loop 4 (Figure S2C, dotted green), a likely less flexible area. In addition, in PorB/VD1-4, the position of VD1 and VD4 was inverted compared to MOMP (VD4 was transferred into PorB loop 4, which precedes VD1 in PorB loop 5); such an inverted order likely generated a completely different epitope interface. This could explain, in part, the scarcer cross-reactivity of anti-MOMP antibodies with PorB/VD1-4 (see Figure 5C), where it is possible that only linear epitopes are recognized. While a similar observation may reasonably apply to the VD1–VD4 interface in PorB/VD1-2-4 (Figure S2D, dotted teal and green, respectively), our cartoon model prediction suggested that the VD4 residues might be more accessible because of the potentially different orientation of the nearby region (Figure S2D, green arrow). None of the residues at the MOMP VD1–VD2–VD4 interface (Figure S2A, teal, green and pink, respectively) were predicted to be adjacent on the structural surface of PorB/VD1-2-4 (Figure S2C, dotted teal, green and pink, respectively). However, despite not being close to each other, it cannot be excluded that these flexible and oppositely charged areas rearrange themselves spatially to allow a more dynamic loop convergence and recreation of a VD1–VD2–VD4 epitope region more similar to that of MOMP. We concluded that the original MOMP conformational epitope is only partly replicated within the PorB/VD antigens, and that new ones are also generated.

The antibody cross-reactivity was also examined against PorB in sera from both mouse strains. Figure S3A,B show that immunization with the PorB/VD antigens induced comparable levels of antibodies that recognized PorB. This suggested that replacing two or even three PorB loops with MOMP regions did not prevent the induction of antibodies against the PorB core or other loops. Reciprocal cross-reactivity confirmed that the PorB/VD antigens were recognized by anti-PorB mouse sera (Figure S3C,D). Although no epitope analysis or comparisons were carried out for PorB, it is likely that some PorB epitopes may also be altered by the loop replacement.

#### 3.3.3. Antibody Cross-Reactivity with *C. trachomatis* Whole Organisms

**Figure 6:**
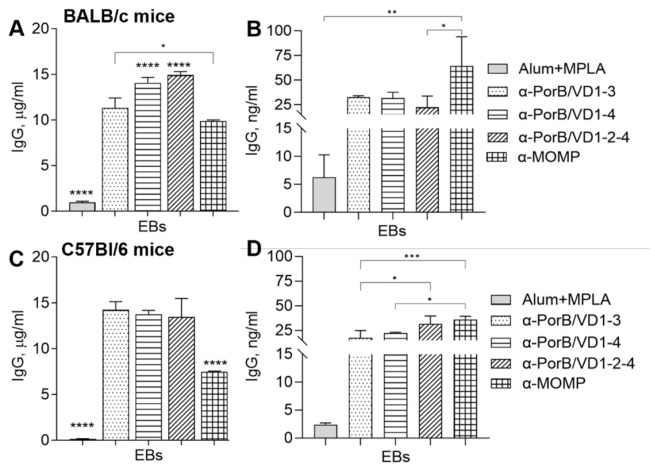
Total IgG antibody cross-reactivity with whole bacteria. IgG (μg/mL ± SD) against *Ct* serovar F EBs in pooled sera from (**A**) BALB/c mice and (**C**) C57Bl/6 mice. Alum+MPLA sera, gray bars. Anti-PorB/VD1-3 sera, dotted bars. Anti-PorB/VD1-4, dashed bars. Anti-PorB/VD1-2-4 sera, striped bars. Anti-MOMP sera, squared bars. Sera were tested in triplicate. IgG (ng/mL ± SD) in pooled vaginal lavages from (**B**) BALB/c mice and (**D**) C57Bl/6 mice as above. Lavages were tested in triplicate. *, **, ***, and **** *p* significant by one-way ANOVA with Tukey’s multiple comparisons test.

The ability of anti-PorB/VD antibodies to recognize MOMP in whole bacteria was examined by ELISA against *Ct* serovar F elementary bodies (EBs). Sera from both mouse strains strongly cross-reacted with the EBs, showing high levels of IgG antibodies specific for MOMP (Figure 6A,C). This suggested that conformational epitopes might be presented and/or recognized more dynamically when MOMP is in its natural state within the EBs compared to the purified recombinant protein. Cross-reactive antibodies against EBs were also detected in vaginal lavages from the PorB/VD-immunized mice (Figure 6B–D), although at a lower level than in sera.

The serum IgG antibody subclasses against EBs were also evaluated. The Th1:Th2 ratio confirmed a generally Th1-skewed response in C57Bl/6 mice, with IgG2c/IgG1 ratios of 1.2 ± 0.16 (PorB/VD1-3), 1.7 ± 0.01 (PorB/VD1-4), 4.3 ± 0.55 (PorB/VD1-2-4) and 1.56 ± 0.13 (MOMP). In BALB/c mice, the IgG2a/IgG1 ratio were 1.07 ± 0.09 (PorB/VD1-3), 0.5 ± 0.05 (PorB/VD1-4), 0.98 ± 0.04 (PorB/VD1-2-4) and 0.21 ± 0.01 (MOMP), suggesting a predominantly Th2-skewed response. No IgA production was detected in the sera or vaginal lavages from BALB/c mice, and a very small, but statistically significant, increase in the serum IgA antibodies to EBs in C57Bl/6 mice immunized with PorB/VD1-3 and PorB/VD1-4 compared to the adjuvant control group, and in vaginal lavages from all the immunization groups in these mice (data not shown).

#### 3.3.4. Neutralization Titers

**Figure 7:**
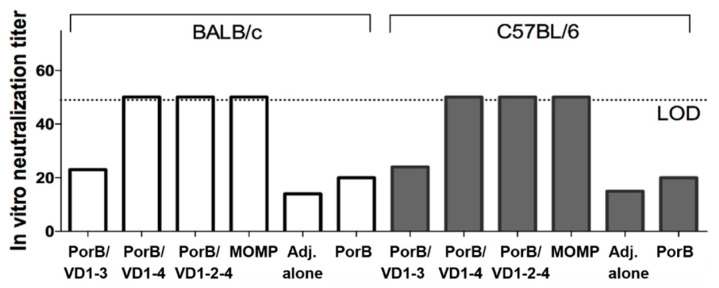
Antibody neutralization titers. Pooled serum samples from BALB/c and C57BL/6 mice (two weeks after the last immunization) were incubated with *Ct* serovar F (1 × 10^4^ IFU) for 45 min. at 37 °C prior to centrifugation onto HeLa-229 cell monolayers in duplicate wells. *Ct* IFUs were detected using a sandwich ELISA with mAb-E4 and counted by light microscopy. Neutralization was defined as a ≥50% decrease in the IFU numbers compared to incubation with control sera from non-vaccinated mice. LOD = limit of detection.

The levels of *Ct* serovar F-specific neutralizing titers induced by the vaccination were measured in sera collected after the last immunization. As shown in Figure 7, PorB/VD1-4, PorB/VD1-2-4 and MOMP elicited the same levels of neutralizing titers in both BALB/c and C57Bl/6 mice. In contrast, PorB/VD1-3 or PorB alone did not induce significant levels of neutralization titers above the sera from mice immunized with Alum+MPLA alone (negative control).

#### 3.3.5. Serum Cytokines

**Figure 8:**
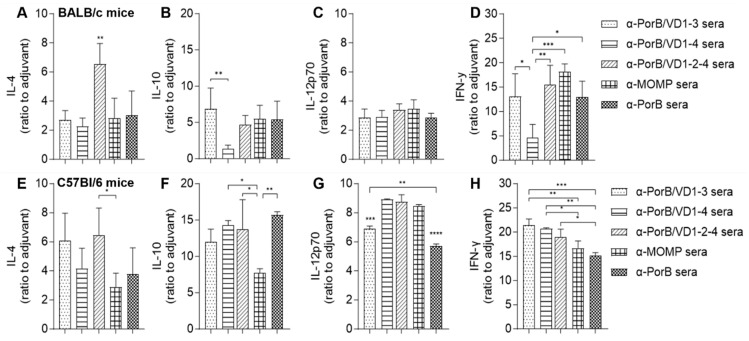
Serum cytokines. (**A**–**E**) IL-4, (**B**–**F**) IL-10, (**C**–**G**) IL-12p70 and (**D**–**H**) IFN-γ in pooled sera from BALB/c mice or C57Bl/6 mice immunized with PorB/VD1-3 (dotted bars), PorB/VD1-4 (dashed bars), PorB/VD1-2-4 (striped bars), MOMP (squared bars) or PorB (checkered bars). Sera were tested in triplicate. Cytokines (pg/mL ± SD) were normalized to sera from mice immunized with Alum+MPLA and expressed as ratio ± SD. *, **, *** and **** *p* significant by one-way ANOVA with Tukey’s multiple comparison test.

For a more complete analysis of the humoral and cellular responses elicited by the PorB/VD antigens, the serum cytokine profile in BALB/c and C57Bl/6 mice was examined. The levels of Th1 cytokines (IL-12p70 and IFN-γ) and Th2 cytokines (IL-4 and IL-10) were quantified and normalized to the levels induced by immunization with the adjuvants alone. In sera from both mouse strains, immunization with PorB/VD1-2-4 induced significantly high IL-4 levels (Figure 8A,E, dashed bars), and this cytokine was also elevated by immunization with PorB/VD1-3 in C57Bl/6 mice (Figure 8E, dotted bar). PorB/VD1-4 induced the lowest IL-10 levels in BALB/c mice (Figure 8B, striped bar), but this was significantly higher in C57Bl/6 mice, along with elevated IL-10 levels induced by all the antigens (Figure 8F). The IL-12p70 levels induced in BALB/c mice was comparable among the antigens (Figure 8C), and a similar trend, but with elevated levels, was observed in C57Bl/6 mice (Figure 8G). The IFN-γ levels induced by PorB/VD1-4 were the lowest in BALB/c mice (Figure 8D) and, similar to IL-10, this was not observed in C57Bl/6, where all three PorB/VD antigens induced higher IFN-γ than in BALB/c mice (Figure 8H).

**Figure 9:**
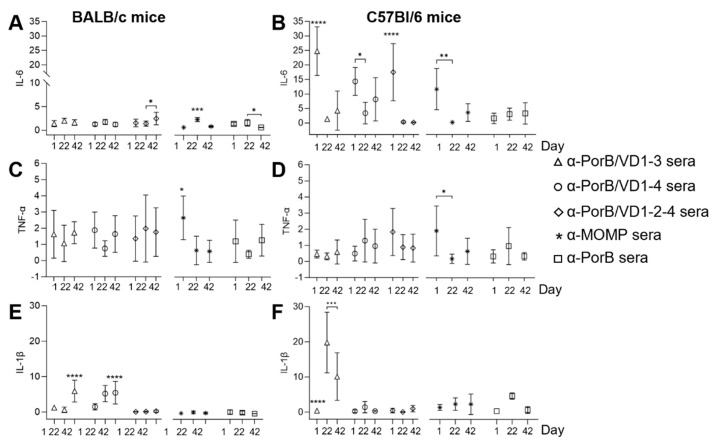
Inflammatory cytokines. (**A**,**B**) IL-6, (**C**,**D**) TNF-α and (**E**,**F**) IL-1β in pooled sera from BALB/c mice and C57Bl/6 mice 24 h after each immunization. Sera were tested in triplicate. Cytokines (pg/mL ± SD) were normalized to sera from mice immunized with Alum+MPLA and expressed as ratio ± SD. PorB/VD1-3, triangles. PorB/VD1-4, circles. PorB/VD1-2-4, diamonds. MOMP, asterisks. PorB, squares. *, **, ***, and **** *p* significant by one-way ANOVA with Tukey’s multiple comparison test.

To gain a sense of the potential inflammatory responses induced by the PorB/VD antigens, IL-6, TNF-α and IL-1β were evaluated in sera collected 24 h after each immunization and normalized to the levels in the adjuvant alone sera. In BALB/c mice, IL-6 was low overall and comparable after each immunization, with only some small, although statistically significant, differences for PorB/VD1-2-4, MOMP and PorB (Figure 9A). In C57Bl/6 mice, IL-6 was transiently elevated after the first immunization with all three PorB/VD antigens and MOMP, but it decreased after the second and third immunization (Figure 9B). The TNF-α levels were similar in sera from both mouse strains, with only a statistically significant increase after the first immunization with MOMP (Figure 9C,D). In BALB/c mice, the IL-1β levels were elevated after the second and/or third immunization with PorB/VD1-3 and PorB/VD1-4 (Figure 9E), and in C57Bl/6 mice, only with PorB/VD1-3 (Figure 9F). These results indicated that C57Bl/6 mice were potentially more prone to inflammation than BALB/c mice.

#### 3.3.6. Splenocytes Proliferation and Cytokine Response

**Figure 10:**
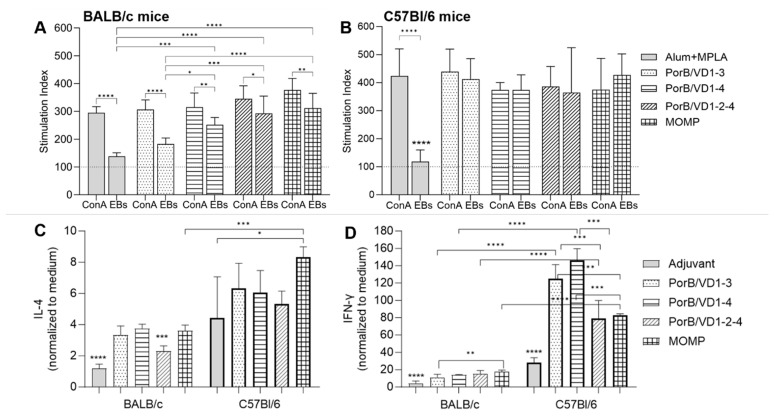
Splenocyte proliferation and cytokine secretion. Proliferation of splenocytes from (**A**) BALB/c mice or (**B**) C57Bl/6 mice stimulated with ConA (10 µg/mL) and EBs (10 µg/mL) for 72 h was normalized to stimulation with medium alone (negative control) and expressed as the Stimulation Index (SI) ± SD from two independent experiments in triplicate. Alum+MPLA, gray bars. PorB/VD1-3, dotted bars. PorB/VD1-4, dashed bars. PorB/VD1-2-4, striped bars. MOMP, squared bars. *, **, and *** *p* significant by two-way ANOVA with Tukey’s multiple comparison test. (**C**) IL-4 and (**D**) IFN-γ in supernatants from stimulated splenocytes from BALB/c mice (thin line) and C57Bl/6 mice (thick line). Cytokines (pg/mL ± SD) were normalized to the medium alone (negative control) and expressed as ratio ± SD. *, **, ***, and **** *p* significant by one-way ANOVA with Tukey’s multiple comparison test.

The cellular responses were examined by measuring the proliferation in vitro and cytokine production by splenocytes from the immunized mice in response to stimulation with *Ct* EBs. The splenocytes were incubated with EBs (10 μg/mL), with medium alone (negative control) or concanavalin A (ConA, 10 μg/mL) (positive control) for 72 h. Proliferation was assessed spectrophotometrically using the MTT chromogenic substrate and reported as the Stimulation Index [(average cell proliferation of stimulated cells/average cell proliferation of the negative control) × 100] [54]. The splenocytes from all the immunization groups in both mouse strains proliferated similarly in response to non-specific stimulation with ConA (Figure 10A,B). Incubation with EBs did not induce significant proliferation of splenocytes from mice immunized with the adjuvant alone (Figure 10A,B, gray bars). In BALB/c mice, EB stimulation induced significantly less proliferation of splenocytes from PorB/VD1-3-immunized mice (Figure 10A, dotted bar) than immunization with PorB/VD1-4, PorB/VD1-2-4 and MOMP (Figure 10A, dashed, striped and squared bars). Furthermore, the EB-induced proliferation was lower than that induced by ConA in these mice. The splenocytes from all the immunization groups in C57Bl/6 mice proliferated in response to EBs, with SI values higher than those in BALB/c and comparable to ConA (Figure 10B), suggesting stronger proliferative responses in these mice.

Secretion of IL-4 and IFN-γ in the supernatants from the stimulated splenocytes was evaluated by ELISA, and the cytokine levels were normalized to the negative control (medium alone). In BALB/c mice, the IL-4 production induced by EB stimulation was significantly higher in cells from the immunized groups than the adjuvant alone group (Figure 10C, thin line bars), and slightly lower for the PorB/VD1-2-4 group than the other groups (Figure 10C, striped bar, thin line). In C57Bl/6 mice, IL-4 induced by immunization with adjuvant alone was elevated and only statistically significantly lower than that from the MOMP group (Figure 10C, squared bar, thick line). The latter was also significantly higher than the BALB/c MOMP-immunized group (Figure 10C, squared bars, thin and thick lines, respectively). A starker difference was observed for IFN-γ production between the immunization groups and both mouse strains, where EB stimulation of the splenocytes from C57Bl/6 mice was much higher than BALB/c mice (Figure 10D). Significant differences were also observed among the immunization groups: PorB/VD1-3 and PorB/VD1-4 significantly higher IFN-γ levels in C57Bl/6 mice (Figure 10D, dotted and dashed bars). These results indicated that immunization with the PorB/VD antigens with Alum+ MPLA as an adjuvant led to a stronger Th1-biased cellular response in C57B/6 mice than in BALB/c mice. The IL-4 and IFN-γ production induced by splenocyte stimulation with ConA was comparable among the groups in both mouse strains (not shown).

## 4. Discussion

In a previous proof-of-concept study using *Cm* MOMP-based constructs, we reported that the PorB/VD1-3, PorB/VD1-4 and PorB/VD1-2-4 antigens induced protective immune responses against *Cm* respiratory infection [42,43]. With the goal of generating a MOMP-based *Ct* vaccine, we developed equivalent constructs displaying the corresponding regions of the *Ct* serovar F MOMP. As a premise for the immunological characterization of these antigens, we used C57Bl/6 and BALB/c mice (known to have biased Th-1 and Th-2 responses, respectively) to ensure that the vaccine constructs will be effective in a large segment of the population. Although only partly replicating the genetic variability and range of immune responses in humans, mice with different genetic backgrounds can also provide information about potential detrimental effects and hyper-sensitivity reactions to vaccination. Adverse effects in vaccine development are multifactorial and can be due to individual factors, i.e., the adjuvant type and/or dosage, the antigen itself, or to the complexity of the vaccine formulation and the intrinsic immune response of the vaccinated subject (humans or animal models) [64].

A preliminary structural characterization of the constructs supported the expectation that the secondary and tertiary structures of the antigens mimicked those of the original PorB carrier protein. Analysis of the protein charges indicated local loop charge changes that could influence electrostatic interactions among the replaced loops, and potentially, their spatial positioning. For example, a strong electrostatic attraction (or repulsion) between loops 5 and 7 could be among the reasons why PorB/VD1-3 showed a high monomer content by non-denaturing electrophoresis. Our previous observation that mutations in these loops affected the ability of PorB to induce TLR2 signaling would support the notion that loops 5 and 7 have a relevant function [47]. Although local loop charge changes may affect nearby loops and possibly have a long-range electrostatic effect on more distant loops [65], whether this has an impact on the proteins’ trimeric structure remains speculative and can only be verified by further structural studies. Nevertheless, the secondary structure predictions suggested that neither the β-barrel core, the α-helix content nor the percent of random coil state was significantly different among the original PorB and the PorB/VD antigens.

The immune responses to the vaccine antigens were explored in BALB/c and C57Bl/6 mice using Alum+MPLA, two adjuvants approved for human use (individually and combined (AS04) [66]), to favor the induction of antibody responses and a Th-1-skewed immunity relevant for a *Chlamydia* vaccine [67]. Robust systemic and mucosal antigen-specific IgG responses were elicited by the three constructs, comparable to our previous results with the *Cm*-MOMP-based antigens [42], with PorB/VD1-3 appearing to be the most immunogenic. As expected, based on the IgG2/IgG1 ratio, the C57Bl/6 mice mounted stronger Th1-biased responses than the BALB/c mice. Anti-PorB/VD antibodies cross-reacted with MOMP, and reciprocal cross-reactivity was also observed between the PorB/VD antigens and anti-MOMP mouse sera, although with a lower specificity than for MOMP. This was not surprising because the PorB/VD antigens only contain limited regions of MOMP. Since PorB/VD1-3 was the most cross-reactive antigen, it is possible that the position and/or presentation of VD1 and VD3 in this construct was a closer replica of the original epitope in MOMP. Analysis of the predicted surface-exposed MOMP B-cell epitope suggested the different spatial positioning of the corresponding regions within PorB/VD1-4 and PorB/VD1-2–4, which may explain, in part, the lower specificity of the anti-MOMP antibodies for these constructs. Although speculative in nature, this is in agreement with our previous observation for the *Cm* MOMP-based antigens: IgG in sera from mice immunized with PorB/VD1-3 recognized well both VD1 and VD3 peptides, anti-PorB/VD1-4 antibodies only recognized well VD1 peptides, and anti-PorB/VD1-2-4 antibodies recognized well VD1 and VD2 peptides, while it did not recognized well VD4 peptides [43]. Revising the cloning strategy could improve the immune recognition of VD4 and more faithfully replicate original regions of the MOMP surface conformational B-cell epitope. Regardless of the PorB/VD antigens’ structural implications or the immune responses detected against the purified proteins, anti-PorB/VD IgG antibodies cross-reacted with the whole *Ct* serovar F EBs, recognizing MOMP regions within the bacterial membrane. Considering that the antibody recognition of a purified protein can be different compared to when such a protein is within the bacterial membrane [68], our results are encouraging. The stronger IgG responses induced by PorB/VD1-3, however, did not parallel the neutralizing antibody levels, while the anti-PorB/VD1-4 and anti-PorB/VD1-2-4 antibodies had a significant neutralizing activity, comparable to that of anti-*Ct* F MOMP. These results are consistent with the neutralizing ability of antibodies directed against MOMP VD1 and VD4 shown in other studies [28,40,62].

Protection against *Chlamydia* infection requires not only neutralizing antibodies but also IFN-γ-producing CD4+ T-cells [10]. We reported the proliferation of splenocytes from the immunized mice in response to re-stimulation with *Ct* serovar F EB, suggesting antigen-specific responses. Proliferation of cells from BALB/c mice was accompanied by the secretion of lower IFN-γ than in C57Bl/6 mice in the T-cell recall assays, supporting more robust and Th1-biased responses in the latter mouse strain. It is interesting that, despite the anti-PorBVD1-3 antibodies not neutralizing, immunization with this antigen also led to high IFN-γ production by splenocytes in response to EB stimulation; it will be important to determine how these immunological findings correlate with protection in vaccination-challenge experiments.

A vaccine that elicits high IFN-γ production by CD4+ T-cells may also be more reactogenic, which can be evaluated by monitoring the inflammatory mediators. The early and transient IL-6 response to PorB/VD1-3 and MOMP observed in C57Bl/6 mice, and the later IL-1β response, may merit additional investigation. These cytokines are considered pyrogenic [64,69,70], and their production in mice could mimic fever that is sometimes observed after vaccination in humans. A transient sub-cutaneous swelling at the site of injection was observed, and in future experiments, the local levels of inflammatory cytokines should be measured (i.e., in the muscle tissue) to evaluate the adverse effects possibly consistent with local pain at the site of injection that can be experienced by some individuals with different vaccines [64].

## 5. Conclusions

Our study supports the PorB/VD antigens as a novel set of potential candidates for a vaccine against *Chlamydia*. Their preliminary structural characterization confirmed that they retained the main features of the PorB carrier protein, and immune profiling indicated that they induced robust and cross-reactive antigen-specific responses against *Ct*. Despite PorB/VD1-3 being the most immunogenic construct, a correlation between the IgG antibody levels and the neutralizing activity against *Ct* could not be established for this antigen. In contrast, anti-PorB/VD1-4 and anti-PorB/VD1-2-4 antibodies were neutralizing against *Ct* serovar F. In addition to humoral responses, immunization with the PorB/VD antigens also induced cellular responses. Proliferation and production of IFN-γ by splenocytes from the immunized mice was observed following *Ct* re-stimulation in vitro, particularly in C57Bl/6 mice. These results confirmed the stronger, Th1-biased anti-chlamydial immune response in these mice compared to BALB/c mice. C57Bl/6 mice were also more prone to inflammation induced by vaccination, which should be further investigated in future vaccine safety studies. Our findings provide a promising foundation for the development of a recombinant *Ct* vaccine capable of inducing comprehensive immune responses. Ultimately, the protective ability of the PorB/VD antigens against *Ct* infection can only be evaluated in a mouse model of vaginal challenge. Subsequent studies can then focus on optimizing the antigen design for enhanced efficacy; for example, modifications to the structure of low-level protective constructs could be tested, and expanding studies of the tertiary/quaternary structure of MOMP in chlamydial EB could significantly facilitate the design of protective antigens.

## 6. Patents

P. Massari, Madico G., de la Maza L.M. Methods and compositions for vaccinating a subject for a sexually transmitted pathogen. U.S. Patent Application15/448,767. 3 March 2017.

## Figures and Tables

**Table 1:** *C. trachomatis* F MOMP amino acid sequence and charge of regions swapped with PorB loops.

Sequence ^a^	Net Charge	PorB Loop Replaced	Charge Change
* 292 **K**WSRASFDSDTIRIAQPRLVTPVVDITTLNPTIAGCGSVAGANTEGQISD**T** 342	−1 (+4/−5)	4	+1 **→** −1
72**R**VLKTDVNKEFEMGEALAGASGNTTSTLSKLVERTNPAYGKHM**Q** 115	+2 (+7/−5)	5	+1 **→**+2
140**L**GATSGYLKGNSASFNLVGLFGDGVNATKPAADSIPNVQLN**Q**181	0 (+2/−2)	6	−1 **→** 0
235**F**TINKPKGYVGKEFPLDLTAGTDAATGTKDASI**D**268	−1 (+4/−5)	7	+1 **→** −1

^a^ Anchor residues, bold red. * T342.

**Table 2:** Secondary structure predictions with SOPMA (self-optimized prediction method with alignment).

	PorB	PorB/VD1-3	PorB/VD1-4	PorB/VD1-2-4
**α-helix** (%)	16.04	16.25	16.24	14.32
**β-sheet** (%)	24.53	25.34	26.29	25.06
**Random coil** (%)	59.43	58.40	57.47	60.62

**Table 3:** Th1:Th2 antibody subclasses ratio (μg/mL ± SD).

	PorB/VD1-3	PorB/VD1-4	PorB/VD1-2-4	MOMP	PorB
**BALB/c mice ^a^**	0.59 ± 0.04	0.96 ± 0.13	0.14 ± 0.02	1.2 ± 0.4	1.07 ± 0.3
**C57Bl/6 mice ^b^**	2.8 ± 0.22 **^c^**	1.9 ± 0.22 **^c^**	0.25 ± 0.02 **^c^**	1.13 ± 0.03	0.86 ± 0.13

**^a^** IgG2a/IgG1; **^b^** IgG2c/IgG1; **^c^** significant vs BALB/c by unpaired *t* test.

## Data Availability

The data can be shared up on request.

## Supplementary Materials

The following figures and tables are available online at https://www.mdpi.com/article/10.3390/vaccines12070789/s1. Figure S1: Amino acid sequence of PorB/VD hybrid proteins. Figure S2: Epitope predictions. Figure S3: Total IgG antibody cross-reactivity with PorB. Table S1. Predicted MOMP linear B-cell epitopes within the regions swapped with PorB loops.
