# Supplementary material for: Structural Assessment of Chlamydia trachomatis Major Outer Membrane Protein (MOMP)-Derived Vaccine Antigens and Immunological Profiling in Mice with Different Genetic Backgrounds
Source: Vaccines (Basel). 2024 Jul 18;12(7):789. doi: 10.3390/vaccines12070789 (PMC11281497; doi:10.3390/vaccines12070789)
Supplement: Supplementary file 1 [file vaccines-12-00789-s001.zip › vaccines-3092519-supplementary.pdf]

**A) PorB/VD1-3**

DVTLYGTIKA GVETYRTVKH TDGKVTEVKT GSEIADFGSK IGFKGQEDLG NGLKAIWQLE
QNASIAGTDS GWGNKQSFIG LKGGFGTVRA GNLNSILKST GDNVNAWESG KATEDVLQVS
KIGAPEHRYA SVRYDSPEFA GFSGSVQYAP KDNSGKNGES YHVGLNYQNS GFFAQYAGLF
Q**R**VLKTDVNK **EFEMGEALAG** **ASGNTTSTLS** **KLVERT**NPAY GKHM**Q**VHRLV GGYDNDALYA
SVAVQQQDAK LTDASNSHNS QTEVAATVAY RFGNVTPRVS YAHG**F**TINKP KGYVG**KEFPL**
**DLTAGTDA**AT GTKDASI**D**QV VVGAEYDFSK RTSALVSAGW LQEGKGAGKT VSTASTVGLR
HKF

**B) PorB/VD1-4**

DVTLYGTIKA GVETYRTVKH TDGKVTEVKT GSEIADFGSK IGFKGQEDLG NGLKAIWQLE
QNASIAGTDS GWGNKQSFIG LKGGFGTVRA GNLNSILKST GDNVNAWESG KATEDVLQVS
KIGAPEHRYA SVRYDSPEFA GFSGSVQYAP **K**WSRASFDSD TIRIAQPR**LV** **TPVVDITTLN**
**PTIAGCGSVA** **GANTEGQIS**D **T**GESYHVGLN YQNSGFFAQY AGLFQ**R**VLKT DVNK**EFEMGE**
**ALAGASGNTT** **STLSKLVERT** NPAYGKHM**Q**V HRLVGGYDND ALYASVAVQQ QDAKLTDASN
SHNSQTEVAA TVAYRFGNVT PRVSYAHGFK GTVAKADGDN RYDQVVVGAE YDFSKRTSAL
VSAGWLQEGK GAGKTVSTAS TVGLRHKF

**C) PorB/VD1-2-4**

DVTLYGTIKA GVETYRTVKH TDGKVTEVKT GSEIADFGSK IGFKGQEDLG NGLKAIWQLE
QNASIAGTDS GWGNKQSFIG LKGGFGTVRA GNLNSILKST GDNVNAWESG KATEDVLQVS
KIGAPEHRYA SVRYDSPEFA GFSGSVQYAP **K**WSRASFDSD TIRIAQPR**LV** **TPVVDITTLN**
**PTIAGCGSVA** **GANTEGQIS**D **T**GESYHVGLN YQNSGFFAQY AGLFQ**R**VLKT DVNK**EFEMGE**
**ALAGASGNTT** **STLSKLVERT** NPAYGKHM**Q**V HRLVGGYDND ALYASVAVQQ QDAK**L**GATSG
YLKGNSASFN LVGLFG**DGVN** **ATKPAADSIP** **NVQLNQ**TEVA ATVAYRFGNV TPRVSYAHGF
KGTVAKADGD NRYDQVVVGA EYDFSKRTSA LVSAGWLQEG KGAGKTVSTA STVGLRHKF

**Figure S1. Amino acid sequence of PorB/VD hybrid proteins. A)** PorB/VD1-3: PorB loop 5 (teal) was replaced with MOMP loop 2 (VD1, bold) and adjacent CD residues (dotted underlined); PorB loop 7 (blue) was replaced with MOMP loop 5 (VD3, bold) and adjacent CD residues (dotted underlined). **B)** PorB/VD1-4: PorB loop 5 replaced as above, and PorB loop 4 (green) was replaced with MOMP loop 7 (VD4, bold) and adjacent CD residues (dotted underlined). **C)** PorB/VD1-2-4: PorB loops 4 and 5 as above; PorB loop 6 (pink) was replaced with MOMP loop 3 (VD2, bold) and adjacent CD residues (dotted underlined). Anchor residues, bold red. In **B** and **C**, MOMP T342 replaced PorB N176 (red star).

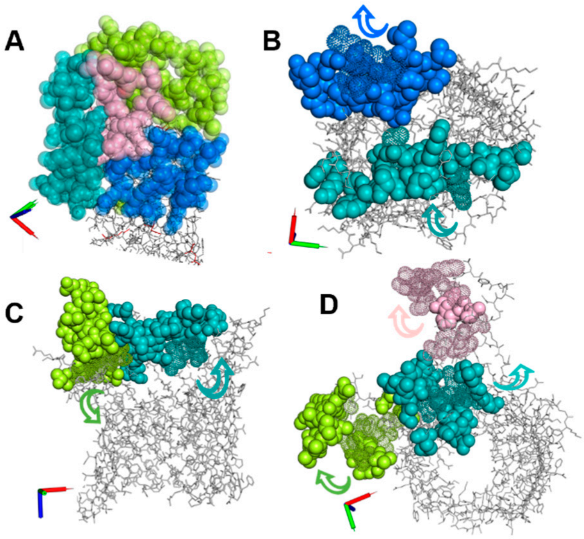


**Figure S2. B-cell epitope predictions. A)** Cartoon model of the predicted surface-exposed conformational B-cell epitope of MOMP obtained with ElliPro and rendered with PyMOL. VD1 residues part of the predicted conformational B-cell epitope are shown in teal, VD2 residues in pink, VD3 residues in blue and VD4 residues in green. The x-y-z axis indicate the model orientation. **B)** Cartoon model of the MOMP B-cell epitope residues transferred into PorB/VD1-3, **C)** PorB/VD1-4 and **D)** PorB/VD1-2-4. The dotted spheres indicate the position of the interfacing residues in MOMP **(A)**, and the arrows indicate their predicted spatial orientation.

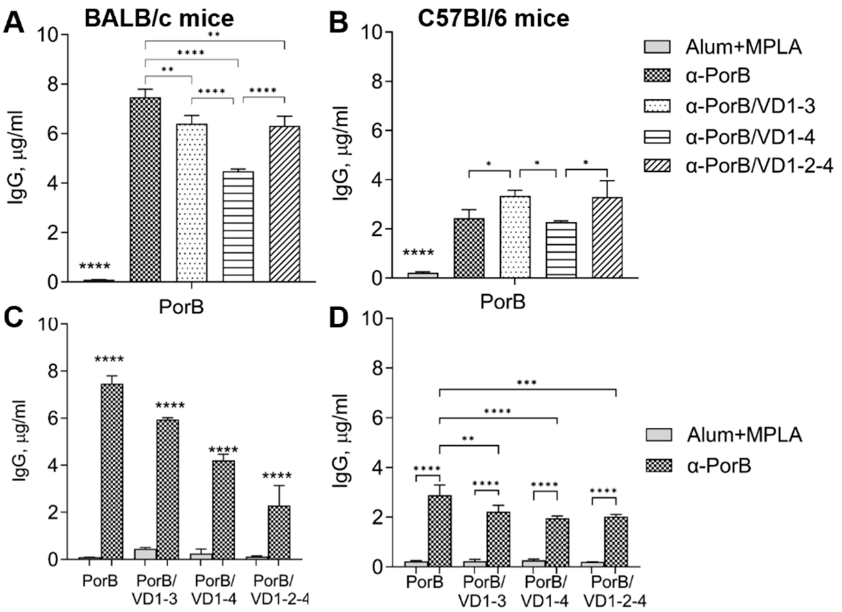


**Figure S3. Total IgG antibody cross-reactivity between PorB and PorB/VDs.** IgG (µg/ml ± SD) against PorB in pooled sera from **A)** BALB/c mice and **B)** C57Bl/6 mice. Alum+MPLA sera, gray bars. Anti-PorB, checkered bars. Anti-PorB/VD1-3 sera, dotted bars. Anti-PorB/VD1-4 sera, dashed bars. Anti-PorB/VD1-2-4 sera, striped bars. **C)** IgG against PorB, PorB/VD1-3, PorB/VD1-4 or PorB/VD1-2-4 in pooled sera from BALB/c mice and **D)** C57Bl/6 mice immunized with PorB (checkered bars) or Alum+MPLA (gray bars). Sera were tested in triplicate. *, **, ***, **** p significant by one-way ANOVA with Tukey's multiple comparisons test .

**Table S1.** Predicted linear B-cell epitopes within the regions of MOMP swapped with PorB loops.

| Sequence | VD | Color |
|---|---|---|
| 78 VNKEFEMGEALAGASGNTTSTLSKLVERTN 108 | 1 | Teal |
| 160 GDGVNATKPAADSI 174 | 2 | Pink |
| 245 VGKEFPLDLTAGTDAATGTKD 264 | 3 | Blue |
| 308 RLVTPVVDITTLNPTIAGCGSVAGANTEGQISDT 342 | 4 | Green |
